# The Clinical Society of Maryland

**Published:** 1893-12

**Authors:** H. O. Reik

**Affiliations:** Secretary; 525 N. Howard St.; Baltimore, Md.


### Society Reports.

### THE CLINICAL SOCIETY OF MARYLAND.

BALTIMORE, MD., NOV., 3, 1893.

The 285th Regular meeting was called to order by the President, Dr. J. Edwin Michael.

Dr. Hiram Woods related a case of Cicatricial Closure of the External Auditory Meatus and exhibited the patient.

The patient, a white man, aged 35, during the Fall of '87 became involved in a quarrel with some one residing in the same house. Sometime after, while sleeping, his enemy entered his room and poured sulphuric acid into his left ear. Suffering terribly, he at once sought medical advice from some one near him. Dr, Woods saw him three weeks later and found the canal filled with granulated tissue, foul suppuration going on and the parts too sensitive to bear examination. Chloroform was administered, the canal scraped, and he found that the drum head was gone and the ossicles apparently burned out. Treatment with view of keeping the canal open, adopted. Suppuration continued. Occasionally the patient would suffer with severe pains, which could be relieved by scraping the canal. Gradually contraction of the external portion of the canal took place, and cicatricial tissue formed over meatus. Whenever the pain became great an incision through the cicatrix would permit the flow of pus and give relief. This was the course for two years. Finally the discharge ceased, the meatus closed up entirely, and for four years the patient has had no pain. There is no perception of sound, save for the fork by bone conduction. There is evidently an opening through the external auditory canal, for inflation by Valsalvan Method produces a perceptible motion of the scar tissue at the opening of the canal. Dr. Woods thinks that to have permitted healing to take place there was probably drainage through the Eustachian tube.

DR. N. G. KEIRLE read a very interesting paper entitled PRELIMINARY REPORT OF CERTAIN EXPERIMENTS CONCERNING ASPECTS OF BABIES.

DR. L. McLANE TIFFANY related two cases bearing upon Renal Surgery—one from his own practice and one from that of Dr. Johnson of Richmond, Va.

Case No. 1. Adult, male negro having all the clinical symptoms of pulmonary tuberculosis. Was suffering extreme pain in the bladder, frequent micturition, urine cloudy, no blood. Opened the bladder above the pubis and introduced a tube. There was no relief. One month and a half later Dr. Tiffany operated on the left kidney, which was then painful upon pressure, opened the capsule freely and gave immediate relief. Patient died one month later of tuberculosis.

Case No. 2. J. S. aet., 65 years. Irish. Occupation, carpenter. In early life had followed the water. Was once operated upon for piles, by ligature, and cured. Temperate in habits. Pain in right loin very severe, accompanied by considerable gastric disturbance and sometimes by retraction of right testicle. Tenderness on pressure over right kidney Sounded bladder with negative result. No blood or pus in urine. Performed Nephro-leithotomy, making V shaped incision, apex upward. Kidney appeared very large, but when pierced by needles showed no sign of stone. Punctured points bled freely. A free slit in the capsule the whole length of the kidney was then made, the bleeding ceased at once and patient was much relieved. Symptoms simulating calculus disappeared and recovery took place.

DR. WM. OSLER remarked that renal pain of severe character, apart from calculus, tuberculosis, and cancer is met with in at least two conditions.

The first is so-called "renal crisis" of Dietl, in which in connection with movable kidney there are paroxysms of furious pains, sometimes associated with vomiting. They may come on quite as abruptly as the attacks of renal calculus, and recur with variable frequency. During them the kidney may be found to be swollen and tender. These symptoms of incarceration, as Dietl termed them, have been attributed to twisting of the vessels, causing strangulation and congestion of the kidney, but more likely to a kink or compression at the origin of the ureter, causing a distension of the pelvis, and in some instances a temporary hydronephrosis. The importance of a recognition of this symptom group is really very great, as cases have been mistaken for abdominal tumor, particularly when the kidney can be felt swollen and tender.

Secondly, there are instances of persistent nephralgia associated with the passage of oxalates, possibly, too, with the passage of a highly acid urine in gouty persons. In the case reported by Dr. James Chadwick,* the patient had all the symptoms of calculus, and the diagnosis was corroborated by a number of eminent surgeons. The attacks had recurred for several years and appeared to be associated always with the presence of an extra amount of the oxalate. The operation demonstrated that there was no stone. Possibly an explanation of the cure by incision of the capsule in some instances of severe nephralgia, in which the symptoms simulate stone, and in which on operation no stone has been found, may be found in the fixation of the kidney and prevention of the mobility on which Dietl's crisis depend.

525 N. Howard St. H. O. REIK, M. D., Secretary.

### SOUTHERN SURGICAL AND GYNECOLOGICAL ASSOCIATION.

ABSTRACT OF THE PROCEEDINGS OF THE SIXTH ANNUAL MEETING HELD IN NEW ORLEANS, LOUISIANA, NOVEMBER 14, 15 AND 16, 1893.

### FIRST DAY—MORNING SESSION.

The Association convened in the assembly room of the Medical Department of Tulane University, and was called to order by the President, Dr. Bedford Brown, of Alexandria, Virginia, at 10 a. m.

*Gynæcologioal Society's Transactions, 1889.

